# Pediatric COVID-19 and Diabetes: An Investigation into the Intersection of Two Pandemics

**DOI:** 10.3390/diagnostics13142436

**Published:** 2023-07-21

**Authors:** Silvia Fotea, Cristina Mihaela Ghiciuc, Gabriela Stefanescu, Anca Lavinia Cianga, Cristina Maria Mihai, Ancuta Lupu, Lacramioara Ionela Butnariu, Iuliana Magdalena Starcea, Delia Lidia Salaru, Adriana Mocanu, Tatiana Chisnoiu, Aye Aung Thet, Lucian Miron, Vasile Valeriu Lupu

**Affiliations:** 1 Clinical Medical Department, Faculty of Medicine and Pharmacy, “Dunarea de Jos” University of Galati, 800008 Galati, Romania; 2 Pharmacology, Clinical Pharmacology and Algeziology, Department of Morpho-Functional Sciences II, “Grigore T. Popa” University of Medicine and Pharmacy, 700115 Iasi, Romania; 3 I-st Medical Department, “Grigore T. Popa” University of Medicine and Pharmacy, 700115 Iasi, Romania; 4 Mother and Child Medicine Department, “Grigore T. Popa” University of Medicine and Pharmacy, 700115 Iasi, Romania; 5 Pediatrics, Faculty of General Medicine, Ovidius University, 900470 Constanta, Romania; 6 Faculty of General Medicine, “Grigore T. Popa” University of Medicine and Pharmacy, 700115 Iasi, Romania; 7 III-rd Medical Department, “Grigore T. Popa” University of Medicine and Pharmacy, 700115 Iasi, Romania

**Keywords:** children, COVID-19, diabetes mellitus, SARS-CoV-2, pediatric population, glycemia

## Abstract

Coronavirus disease 2019 (COVID-19) is a complex infectious disease caused by the SARS-CoV-2 virus, and it currently represents a worldwide public health emergency. The pediatric population is less prone to develop severe COVID-19 infection, but children presenting underlying medical conditions, such as diabetes mellitus, are thought to be at increased risk of developing more severe forms of COVID-19. Diabetic children face new challenges when infected with SARS-CoV-2. On one hand, the glycemic values become substantially more difficult to manage as COVID-19 is a predisposing factor for hyperglycemia. On the other hand, alongside other risk factors, high glycemic values are incriminated in modulating immune and inflammatory responses, leading to potentially severe COVID-19 cases in the pediatric population. Also, there are hypotheses of SARS-CoV-2 being diabetogenic itself, but this information is still to be confirmed. Furthermore, it is reported that there was a noticeable increase in the number of cases of new-onset type 2 diabetes among the pediatric population, and the complications in these patients with COVID-19 include the risk of developing autoimmune diseases under the influence of stress. Additionally, children with diabetes mellitus are confronted with lifestyle changes dictated by the pandemic, which can potentially lead to the onset or exacerbation of a potential underlying anxiety disorder or depression. Since the literature contains a series of unknowns related to the impact of COVID-19 in both types of diabetes in children, the purpose of our work is to bring together the data obtained so far and to identify potential knowledge gaps and areas for future investigation regarding COVID-19 and the onset of diabetes type 1 or type 2 among the pediatric population.

## 1. Introduction

The severe acute respiratory syndrome coronavirus 2 (SARS-CoV-2) was first isolated and identified, in Wuhan, China, in 2019 [1]. Many studies suggested that viruses, like enteroviruses (Coxsackie virus B), rotavirus, mumps virus and cytomegalovirus, are potential triggers of Type 1 diabetes mellitus (T1DM) in children and young adults [2]. Currently, SARS-CoV-2 infection also appears to be a potential trigger for the development of diabetes mellitus type 1 and 2 in children, which represents the most frequent chronic metabolic disorder within the pediatric population. In their study, Graff et al. [3] found that among 454 patients identified with SARS-CoV-2 infection, those with diabetes were more prone for admission (aOR, 6.6; *p* = 0.04), and, moreover, data also showed that diabetes mellitus and other cardiovascular comorbidities were identified as major risk factors regarding outcome and mortality in patients with COVID-19. ACE2 is a receptor expressed in various organs, including both exocrine and endocrine tissues of the pancreas. In a manner similar to SARS-CoV, the virus responsible for the 2003 pandemic, SARS-CoV-2 binds to ACE2 receptors through its spike protein. Since the emergence of the SARS-CoV-2 pandemic, diabetes has been acknowledged as a risk factor associated with increased morbidity and mortality in patients with COVID-19. Moreover, recent evidence indicated that COVID-19 may result in poorer outcomes among individuals with pre-existing diabetes, potentially leading to the development of diabetic ketoacidosis (DKA) [4].

Clinical manifestations of COVID-19 are correlated with age according to the data available in the literature [5]. Pediatric patients with no important medical history are susceptible to COVID-19 but usually have a milder course compared to adults [6]. COVID-19 can exhibit a pronounced clinical progression involving acute respiratory distress syndrome (ARDS), accompanied by a localized and systemic surge of cytokines, which can potentially lead to rapid clinical deterioration and failure of multiple organs. Compared to adults who develop respiratory symptoms that can evolve into ARDS, most children do not have respiratory diseases but can develop a life-threatening multisystem inflammatory syndrome (MIS-C). MIS-C is a multi-system inflammatory syndrome that affects individuals aged <21 years with a history of SARS-CoV-2 infection within 4–6 weeks prior to the onset of symptoms presenting with fever, laboratory evidence of inflammation and multisystem (>2) organ involvement, with cardiac and gastrointestinal being the most frequent issues and no alternative plausible diagnoses [7]. In children, MIS-C exhibits similarities to Kawasaki disease, such as febrile illness with inflammation of the blood vessels and possible subsequent consequences of coronary artery aneurysm, conjunctivitis, rash and congestion of the oropharynx [8]. Neurological manifestations, such as headache, meningism, skin hyperesthesia and altered consciousness, were also described in MIS-C [9]. Nevertheless, these findings are non-specific and are found in a vast area of other pediatric infectious diseases [10].

The defects in insulin action and/or secretion are present in diabetes mellitus, which sums up a group of chronic metabolic diseases that imply elevated blood glucose values, mentioning that insulin action and/or secretion disorder may coexist in the same patient [11]. Diabetes can be classified as type 1 diabetes (insulin-dependent diabetes mellitus) and type 2 diabetes (non-insulin-dependent).

### 1.1. T1DM

Type 1 diabetes mellitus (T1DM) occurs when the beta cells in Langerhans pancreatic islets are gradually destroyed by the immune system, leading to a decrease in the body’s insulin production capacity, eventually resulting in no insulin production [12]. If insulin is lacking, the production of hepatic glucose increases and the use of insulin decreases. This leads to a significant breakdown of adipose tissue, ketonemia and successive diabetic ketoacidosis that can be life threatening if not treated [13].

The diagnosis of type 1 diabetes is established based on the association between clinical manifestations, such as polydipsia, polyuria, weight loss, the laboratory findings and the autoantibody positivity, and other clinical signs can include abdominal pain, vomiting, shortness of breath and an altered general status. Diabetes can sometimes be misdiagnosed as asthma (because of Kussmaul respiration) or as an acute abdomen, but these errors are becoming rare with the help of laboratory testing [14]. In the absence of important comorbidities, T1DM and its treatment are known to have multiple complications, the two major ones being diabetic ketoacidosis and hypoglycemia.

Acute hyperglycemia leads to diabetic ketoacidosis, which is an acute life-threatening metabolic complication of diabetes with a mortality rate of 0.5 percent and with almost 40 percent of DKA cases at presentation. Cerebral edema represents the most frequent cause of death among the diabetic population. For the already known cases of diabetes, the rate of this complication is 1 to 8 percent per year. DKA is managed with immediate hospitalization, insulin replacement and rehydration [15,16].

On the other hand, hypoglycemia is a T1DM complication of insulin treatment. Its symptoms are the result of a fall in blood glucose, leading to a range of neurogenic and neuroglycopenic symptoms, including emotional instability and tremor. In critical cases, seizures and unconsciousness may be present, and there are suspicions regarding permanent cognitive sequelae caused by repeated hypoglycemic episodes, which are to be confirmed [17,18]. This condition was reported to exist in approximately 19–37 percent of children and adolescents with the tendency of reduction in the prevalence over time [19]. When severe hypoglycemia occurs, urgent treatment is required and can be effective with the administration of glucagon intravenously, intramuscularly or subcutaneously [20].

Studies describe that SARS-CoV-2 infects [21] pancreatic cells, leading to replication and alteration of the β-cell function precisely and via impairment of insulin secretion. Moreover, SARS-CoV-2 triggers autoimmunity [22], meaning that both autoantibody-positive insulin-dependent and autoantibody-negative diabetes can develop during an infection with the novel coronavirus [23]. 

### 1.2. T2DM

Type 2 diabetes mellitus (T2DM) is a complex, chronic metabolic disease that consists of hyperglycemia as a result of insulin resistance and a variable grade of impairment in insulin secretion due to β-cell dysfunction (lipotoxicity, inborn genetic defect or acquired from glucose toxicity or other mechanisms) [24].

The frequency of type 2 diabetes has increased significantly around the world over the past 20 years [25]. In the pediatric populations, T2DM accounts for less than 50% of all diabetes cases, and there are significant ethnic differences in prevalence all around the world. After adjustment for other demographic characteristics, Pham et al. demonstrated, in their study, that the probability of having type 2 diabetes was double among the Asian population, 65% more likely among Black people, and 17% more likely among people of mixed/other ethnicities [26,27]. The constantly increasing incidence of type 2 diabetes mellitus among children and adolescents is also becoming a source of concern to all those involved in the care of diabetic children [28].

Usually, the debut of T2DM occurs during puberty in high-risk obese adolescents having insulin resistance, knowing that the data regarding the prevalence of T2DM among children under 10 years of age are very limited [29]. Clinically, it presents characteristics of metabolic syndrome, such as arterial hypertension, hyperlipidemia, acanthosis nigricans, fatty liver disease and polycystic ovary disease [26]. The complications of T2DM are of great importance. Arterial hypertension and imbalance of lipids have an impact on the early development of retinopathy, nephropathy and increase the risk of future cardiovascular disease, which is a major cause of morbidity and mortality in adults with T2DM. These comorbidities occur early and progress at an alarming rate in children and adolescents, even in those with efficient metabolic control. Unfortunately, in comparison to the adult population, treatment options for children are limited. Needless to mention, an early initiation of treatment with metformin alongside lifestyle modifications and physical activity for all youth with T2D remains imperative and leads to better compliance [30,31].

In addition, measures, like social distancing, online schooling, increased intake of high-calorie foods, isolation with consequent reduction in physical exercises, leading to a worsening of preexistent obesogenic factors in a population, and health disparities with temporization of seeking medical care, may have all led towards an increased incidence of the disease as the pandemic developed [19].

During the COVID-19 pandemic, diabetic children encountered difficulties regarding dietary habits, physical activity, insulin dose adjustment and, more rarely, access to insulin supply due to the challenging lifestyle changes [32]. Moreover, a significant increase in severe ketoacidosis was discovered in diabetic pediatric cases during the same interval of time [33]. 

To collect studies from 2019 to 2023, the MEDLINE-PubMed database was queried for controlled clinical trials, systematic reviews, randomized placebo-controlled trials and controlled clinical trials. The search used a combination of keywords: “COVID-19” OR “SARS-CoV-2” AND “diabetes mellitus” OR “type 1 diabetes” OR “type 2 diabetes” AND “pediatric” OR “paediatric” OR “children” OR “infant” OR “adolescent”. Additionally, we manually searched the reference lists of the obtained studies. The search was restricted to English-language journals. Our extracted data included study characteristics, participant characteristics (age, diabetes type) and COVID-19 outcomes (severity, onset, diagnosis, complications). Two independent reviewers excluded the review articles, case reports and studies not directly related to our research question. The findings of the included studies were summarized qualitatively, and we highlighted the main outcomes, trends and limitations. 

## 2. Diabetes Mellitus and COVID-19 in Children

### 2.1. T1DM and COVID-19

**Figure 1:**
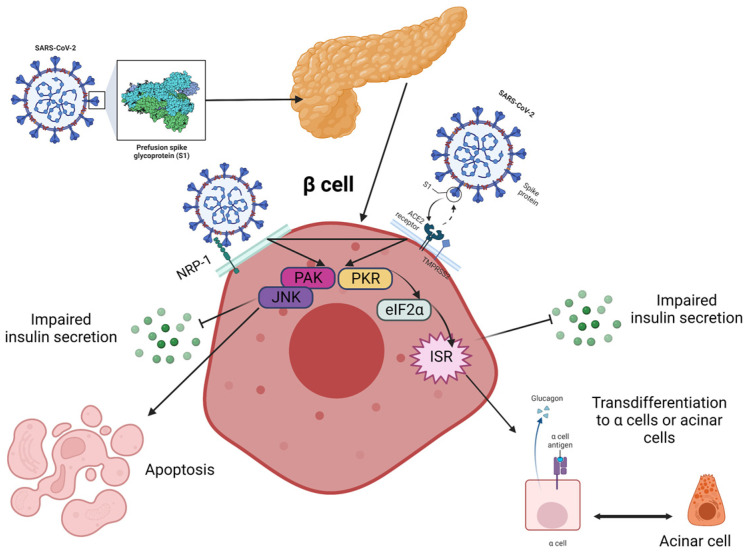
Proposed pathways for the fate of β cells post-COVID-19 are currently being investigated, although it remains unclear how the virus is transmitted to the pancreas and islets. Neurophilin-1 (NRP-1) has higher expression in β cells compared to angiotensin-converting enzyme 2 (ACE2) and could play a critical role in the infection. The virus stimulated β cells via p21-activated kinase (PAK) and c-Jun N-terminal kinase (JNK) and triggered the transformation of β cells into glucagon-producing α-cells or trypsin-producing acinar cells, leading to decreased insulin secretion. This transformation is facilitated through the PKR-eIF2a-mediated integrated stress response (IKR), and these two pathways may interact with each other. The eIF2 is the eukaryotic translation initiation factor 2, and PKR stands for protein kinase R. Created with Biorender.com, adapted after [38].

From the beginning of the pandemic, it was quickly discovered that pre-existing T1DM represented one of the high risk factors for developing severe COVID-19 and related complications. On the other hand, SARS-CoV-2 has been suggested as a potential inducer of new-onset pediatric T1DM [34]. Data available in the literature at this moment suggest that severe acute respiratory syndrome coronaviruses (SARS-CoV-2, for instance) can enter in islet cells via angiotensin-converting enzyme-2 (ACE-2) receptors and cause reversible β-cell damage and transient hyperglycemia. Also, the discovery of SARS-CoV-2 in pancreatic tissue samples taken from deceased individuals, along with the evidence of decreased pancreatic function in both the pediatric and adult population with COVID-19, implies that this virus might harm the β cells of the pancreas and trigger the onset of T1DM through direct infection, inflammatory response and interactions with the renin-angiotensin system (Figure 1). It was proposed that several pathways may be involved, and β cells can be infected through several transmembrane receptors beyond ACE-2, such as neuropilin 1 (NRP-1), transferrin receptor (TFRC) and FES Upstream Region (FURIN). Also, immunofluorescent studies demonstrated a stronger expression of NRP-1 in β cells compared to α cells, and inhibition of this receptor with a specific antagonist reduced the uptake of SARS-CoV-2, suggesting its important role in viral entry. Downstream effects appear to involve the activation of key signaling pathways, such as c-Jun N-terminal kinase (JNK) and p21-activated kinase (PAK), that lead to apoptosis and impaired insulin excretion. Moreover, according to Tang et al., SARS-CoV-2 can lead to a transdifferentiation of β cells into α and acinar cells via the signaling pathway, involving the phosphorylation of protein kinase R (PKR) and eukaryotic translation initiation factor 2 (eIF2), which, in turn, determines integrated stress responses and cellular conversion [35,36,37,38].

From a different perspective, the importance of psychological stress is also not to be neglected as it is known for decreasing insulin sensitivity and increasing insulin resistance and, therefore, may be important in the development or the onset of T1DM. The pandemic and the subsequent lockdown have had biological, economic, psychological and social consequences, and they may have increased the risk of type 1 diabetes during this period [39].

Usually, most pediatric patients with COVID-19 present mild clinical manifestations and have good prognosis. They can either have fever and mild upper respiratory symptoms or no symptoms at all. Also, they may exhibit gastrointestinal symptoms or manifestations related to DKA and may present polyuria, polydipsia, extreme fatigue, elevated temperature, drowsiness, tachypnea, deep breathing, abdominal pain, nausea, vomiting and somnolence with a clinical course that can even progress to coma and life-threatening status [40].

Diabetes and COVID-19 can have immunosuppressive effects, which lead to an increased risk for fungal disease, especially in those patients who receive corticosteroid therapy. Brothers et al. described the clinical findings of a fungal infection with Candida glabrata, a known pathogen in diabetic patients that can give rise to fulminant septic shock due to its increased resistance to antifungal agents in a child with type 1 diabetes [41,42]. 

A case report also described an association between new-onset diabetes and MIS-C with overlapping symptoms from both diseases with typical signs of DKA and associated bilateral conjunctivitis with limbic sparing swollen, bright red with dry mucous membranes, scattered cervical lymphadenopathy, stage 1 acanthosis nigricans and impaired mental status that were identified in the evolution of a patient. The simultaneous presence of both conditions may suggest that COVID-19 may have an impact on both beta cell function and beta cell death by accelerating its course [42]. 

Data published in the literature prove that there is a positive association between COVID-19 and thrombotic disorders, with evidence also suggesting that macroangiopathic processes and complement-mediated inflammation may be involved [43]. For instance, Grigore et al. described the case of cerebral venous thrombosis secondary to SARS-CoV-2 infection in a female teenager [44], whereas Alizadeh et al. presented a case of new-onset diabetes and atypical hemolytic-uremic syndrome, raising the suspicion that SARS-CoV-2 infection may represent an infectious trigger for the patient’s condition [45].

**Figure 2:**
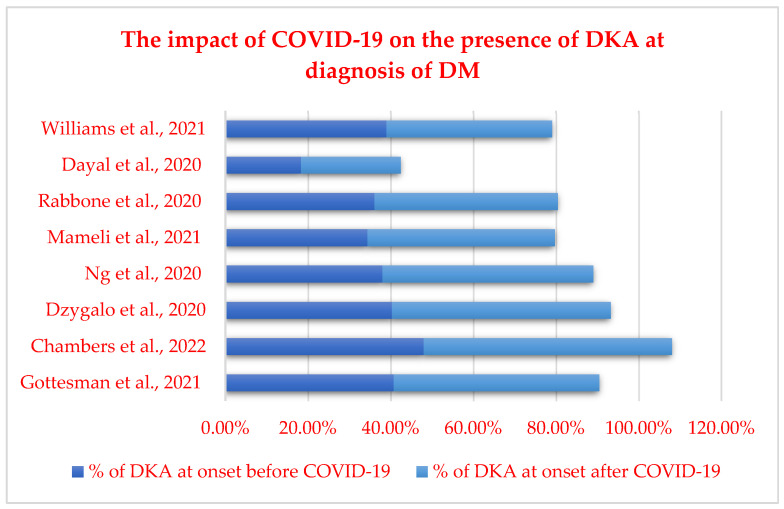
Overview of the studies regarding the impact of COVID-19 pandemic on the presence of DKA at the moment of DM diagnosis [46,47,48,50,51,52,53,54].

In a cross-sectional survey conducted in Italy in November 2020, Rabbone et al. [46] described how the COVID-19 pandemic might have reduced diabetes presentation and the severity of diabetic ketoacidosis by comparing data across two consecutive years (2019 and 2020). Surprisingly, compared to the same period in 2019, the results in 2020 showed a reduction of approximately 20% in new diabetes cases. Similar situations were noticed both in India and the UK [47,48]. A possible explanation would be that along with the lockdown, children were less exposed to seasonal viruses, which are known to be triggers for new-onset type 1 diabetes cases [49,50]. On the other hand, children presenting with DKA had a more aggressive form of DKA in 2020 compared to 2019 (44.3% vs. 35%) [46], and a significant rise in DKA at follow-up was observed during the first wave in many countries with high COVID-19 mortality [51]. A summary of the studies regarding the impact of the COVID-19 pandemic on the presence of DKA at the moment of diabetes mellitus diagnosis is represented in Figure 2.

A study from Germany suggested an increase in the number of cases of diabetic ketoacidosis alongside more severe cases of DKA at diagnosis among the pediatric population during the COVID-19 pandemic, with an incidence of DKA that almost doubled in 2020 compared to the previous year [34]. Additionally, a study conducted in the UK demonstrated an increase of 80% regarding the number of cases of T1DM in children compared to 2019 and 2018, along with a high rate of severe DKA but without delayed presentation [35]. Ho et al. agreed with these facts in their study, which proved that not only the frequency of DKA at the debut of T1DM was more increased during the pandemic (68.2% vs. 45.6%; *p* < 0.001) but the incidence of severe cases of DKA was also more important (27.1% in 2020 vs. 13.2% in 2019; *p* = 0.01) [55]. 

Moreover, there are also results that show that children presenting HbA1c measurement was higher in those presenting during COVID-19 than one year earlier (3.0 ± 1.7 vs. 10.4 ± 3.2%; 119 ± 19 vs. 90 ± 35 mmol/mL; *p* = 0.008) [56]. Alonso et al. even found that higher HbA1c was significantly associated with hospitalization and considered HbA1c as a predictor for hospitalization with COVID-19 [57].

In another study, Trieu et al. showed an important increased incidence of 16.3% of new-onset T1DM in 2020 compared to the same period in 2019, which becomes even more relevant when compared to the same period in the previous two years (2018 and 2019), during which only a 6.5% decrease was observed. These results also apply to patients with T1DM who presented with DKA during 2020, representing 64.3% compared to 56.9% in 2019 and 47.1% in 2018 [58]. Another interesting aspect of the relationship between COVID-19 and children with T1DM is the seasonality, which was evaluated by Kostopoulou et al. and shows an increasing tendency from spring to winter (spring: 9.5% vs. 23.5%, autumn: 23.8% vs. 29.4%, summer: 19% vs. 11.8%, winter: 47.6% vs. 35.3%) [59].

### 2.2. T2DM and COVID-19

**Figure 3:**
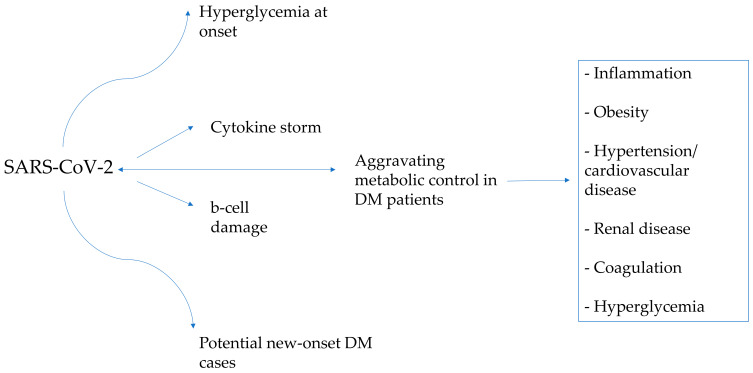
The interplay between diabetes and COVID-19 and how they affect each other.

Statistically, the prevalence of T2D among the pediatric population has significantly risen in recent years [60,61]. The World Health Organization (WHO) reports that in America, Europe and the Eastern Mediterranean, roughly 50% of the population is classified as overweight or obese, with lower rates in Africa and Asia [62]. The COVID-19 pandemic and its consequent circumstances, such as the movement restrictions and the repeated lockdown measures, could have played a substantial role in increasing the number of these patients in the world [63]. The mechanisms that may explain this phenomenon are described in Figure 3. 

The pathways leading to hyperglycemia at the initiation of T1DM/T2DM and hyperglycemia during infection-related exacerbation of metabolic control in children with diabetes may present both convergent and distinct mechanisms. During the onset of diabetes, hyperglycemia results as a consequence of pancreatic beta cell loss/dysfunction with impaired insulin production, whereas during infection-related exacerbation of metabolic control in individuals with diabetes, factors, such as stress hormones, cytokines and the consequent inflammatory response, are responsible for the compromise of the insulin action. Clearly, while some shared pathways may be implicated in hyperglycemia at the debut of diabetes and hyperglycemia during SARS-CoV-2 infection aggravating metabolic control in DM patients, the underlying mechanisms can also exhibit divergence, contingent upon the specific circumstances and individual characteristics. 

Multiple studies have indicated that children and adolescents experienced an abnormal increase in weight during the COVID-19 pandemic, exceeding the expected weight gain for their age [64,65,66,67,68]. In this regard, implementing an integrated approach that promotes physical activity, lifestyle counseling and psychological support is deemed critical [69,70]. Additionally, obesity is considered a risk factor for COVID-19 infection, and patients with obesity who become infected are more susceptible to experiencing severe forms of the disease [71,72,73]. During the pandemic, there were various risk factors, such as online school activities, a more sedentary lifestyle, chronic stress and increased caloric intake, reduced availability and access to sports and physical exercise, increased social isolation and food insecurity, that led to an increased body mass index (BMI) during this period. Recently, the Centers for Disease Control showed in a data analysis that BMI doubled its values during the pandemic compared to a pre-pandemic period among the pediatric population [74]. This is explained by the increase in the number of snacks and a reduced rate of physical exercise during lockdown [75].

Anderson et al. proved, in their systematic review and meta-analysis, that in the initial year of the COVID-19 pandemic, there were consequential rises in weight gain, BMI and a higher incidence of obesity in both adults and children [76].

The same affirmation was stated by Sasidharan Pillai et al. in their retrospective study conducted on new-onset T2DM patients, who found that BMI increased during the COVID-19 pandemic compared to previous years (129% of 95th percentile vs. 141%, *p* = 0.02). Bond et al. discovered that not only did the BMI percentile peak after COVID-19 restrictions, but over the course of the following 21 months, it returned to pre-pandemic levels (β = −0.04 (95% CI −0.13, 0.04)) [77,78].

During the period from April to November 2020, Trieu et al. noticed a 16.3% increase in the occurrence of newly developed T1DM and a 205.3% increase in the occurrence of newly developed T2DM when compared to the same time frame in 2019. In 2019 and 2018, among children who experienced new-onset T1DM, 56.9% and 47.1%, respectively, presented with DKA, whereas in 2020, this percentage rose to 64.3%, a value which exceeded the national average. Within this period, a total of 28 children were diagnosed with both COVID-19 and diabetes. Notably, two cases exhibited significant complications due to COVID-19 and DKA, necessitating the administration of high doses of intravenous insulin over an extended duration [58]. Like many other viral infections, COVID-19 may worsen the already dysregulated glucose metabolism, leading to an increased insulin requirement [79].

Hospitalizations for new-onset type 2 diabetes in children also increased in 2020 compared with the same interval of time in 2019, according to findings presented at the American Diabetes Association Scientific Sessions. 

The data obtained from a hospital in Louisiana indicated a rise in the number of admissions for new-onset T2DM in 2020 (8 cases out of 2964 hospitalizations) accompanied by DKA compared to 2019 (17 cases out of 2729). Additionally, two young individuals met the criteria for hyperosmolar hyperglycemic syndrome in 2020, whereas no cases were observed in 2019. Furthermore, increases were observed in the average levels of HbA1c, glucose and serum osmolality upon admission from 2019 to 2020 [80]. 

On the other hand, despite various studies indicating an increased occurrence of new-onset T2DM and DKA, Lee et al. did not observe similar findings in Korea. The data collected between 2018 and 2020 indicated that the annual incidence of DKA in T2DM patients did not differ notably, nor did the total number of newly diagnosed T2DM patients (24 in 2018, 24 in 2019 and 33 in 2020). Furthermore, they reported no notable changes in BMI and weight parameters between the pre-pandemic and pandemic intervals in patients with T2DM [81]. This contrasting result raises questions and suggests potential regional- or population-specific variations in the relationship between the pandemic and diabetes. Moreover, this study focuses on the Korean population, which may limit its generalizability to other regions and ethnicities. 

## 3. Discussion

The relationship between DM and COVID-19 is bidirectional, as individuals with DM are at a higher risk for worse COVID-19 outcomes due to multiple associated conditions, while SARS-CoV-2 can also cause new-onset diabetes or maintain hyperglycemia in those infected because of its tropism for the β cell. This impairment of β-cell function, coupled with the inflammatory cytokine storm and counter-regulatory hormonal responses, can lead to acute metabolic complications, such as DKA or hyperglycemic hyperosmolar syndrome. The occurrence of new-onset diabetes, hyperglycemia upon admission and acute metabolic deterioration can further exacerbate the severity of COVID-19 outcomes [82].

The purpose of this review was to expose the role of the trigger of COVID-19 on the onset of new DM cases among the pediatric population and to also establish and emphasize the importance of recognizing patients with T1DM and T2DM who are at great risk for severe forms of COVID-19 infection. Most of the studies showed that control of glycemic values in children with diabetes worsened during the initial quarantine period of the pandemic, with children on public insurance affected to a greater proportion than those with private insurance and with Afro-American patients having higher HbA1c than white patients. Also, the cases encountered in clinical practice proved that the management of hyperglycemia in COVID-19 patients may be more difficult [83,84].

Clearly, the COVID-19 pandemic had a major impact on many aspects of human life, including access to healthcare and the delivery of medical services. Thus, simultaneous new-onset T1DM and COVID-19 in a patient from the pediatric field represent a difficult challenge for the medical team, and this is the reason why the field of telehealth should be more explored in the future [85]. A study conducted in Bangladesh even proved that fasting during Ramadan can be attainable for patients with T1DM with adequate counselling and support through telemedicine [86]. Also, the results of a study in Saudi Arabia confirmed the clinical effectiveness of telemedicine in diabetes care during lockdown [87]. However, the drawbacks of telemedicine include the fact that there is a significant number of patients who do not have an internet connection, and there are also some features of in-person encounters and certain interactions that cannot be replicated through virtual spaces, including physical examinations, routine tests and complication screenings [88,89]. Contrary to some opinions, in a cohort on Finnish children admitted to the pediatric intensive care unit for new-onset T1DM and children registered with the national diabetes registry, Salmi et al. proposed that the higher frequency of new-onset T1DM derives, most likely, from the delay in its diagnosis and not because of SARS-CoV-2 infection [90]. The study found that none of the analyzed children diagnosed with T1DM during the pandemic tested positive for SARS-CoV-2 antibodies, suggesting that the increased incidence of T1DM and DKA is unlikely to be directly caused by SARS-CoV-2 infection. The delay in diagnosis may result from changes in parental behavior and healthcare accessibility, a hypothesis that highlights the potential impact of external factors on timely T1DM diagnosis. However, this study was conducted at a single center, which may limit the generalizability of the findings to other settings or populations. Moreover, although the study did not identify any SARS-CoV-2-positive cases among the analyzed children, it is also important to acknowledge that not all children were tested for SARS-CoV-2 antibodies. In consequence, the hypothesis of asymptomatic or undetected cases could not be completely excluded. 

Most of the studies included in this review concluded that a decrease in physical activity along with an unhealthy diet led to global impaired glycemic control and contributed to its aggravation. Many patients declared that their dietary patterns were unhealthier during confinement and had a sedentary lifestyle [4,91]. 

Several studies conducted in the United States, Sweden, China and Italy examined the pre-lockdown, lockdown and post-lockdown periods to assess the impact on glycemic control in children and adolescents with type 1 diabetes during the pandemic. These studies consistently demonstrated that glycemic control did not worsen and, in fact, showed improvement, particularly in the Italian cohort [92,93]. One plausible explanation for this positive outcome might be the implementation of a more stable and slowed-down lifestyle, enabling patients to exert better control over their disease [77,94,95].

Another important aspect is that there are also good results concerning the relationship between the COVID-19-pandemic-related lockdown and diabetes distress. Mianowska et al. showed, in their study, that staying at home was even beneficial for some patients. These children registered a distress decline, most likely due to the fact that it might have been easier for them to control their disease and to avoid school-related distress [96]. This theory is not supported by multiple studies, which showed that the problems described most frequently during the pandemic were represented by sleep disruption, anxiety, depression, eating disorders and parenting stress [97,98,99,100].

An increase in the number of new-onset T1DM and DKA cases was noticed in the United Kingdom and Germany, but on the other hand, a lower incidence of these cases was observed in Italy during the preliminary period of the COVID-19 pandemic. A comprehensive meta-analysis also showed that, in comparison to the period before the COVID-19 pandemic, there was a notable increase in the worldwide incidence of pediatric new-onset T1DM, DKA and severe DKA during the initial year of the pandemic, with percentages rising by 9.5%, 25% and 19.5%, respectively. Furthermore, when comparing the post-pandemic period to pre-pandemic levels, there was a substantial elevation of 6.43% and 6.42% in median glucose and HbA1c values, respectively, among newly diagnosed children with T1DM [101]. The infection caused by SARS-CoV-2 has the potential to induce both hyperglycemia and the occurrence of ketoacidosis, impacting individuals with diabetes as well as those without prior diabetes. This concurrent presentation of a hyperglycemic state and ketoacidosis poses a significant risk for fatal outcomes [102]. Alamuri et al. explored similar aspects in their review, where the findings indicated that DKA occurring in the context of COVID-19 appeared to elevate the risk of mortality, particularly among individuals with newly diagnosed diabetes [103]. The increased rates of new-onset T1DM and T2DM pediatric admissions during the pandemic are congruous with studies suggesting an increased occurrence of new-onset T1DM that may be accelerated by the coexistence of COVID-19. Despite the numerous reports on COVID-19 and T1DM in children, the data on T2DM in children remain insufficient. However, data from retrospective cohorts analyzed by Barrett et al. highlight an important increase (HR = 2.66, 95% CI = 1.98–3.56) in the total number of T1DM and T2DM among patients with COVID-19 compare to those who were not infected [104]. On the other hand, in adult hospitalized patients, those with a prior history of prediabetes who also had COVID-19 exhibited a significantly higher occurrence of incident diabetes mellitus compared to hospitalized patients without COVID-19 and with a history of prediabetes (21.9% vs. 6.02%, with *p* < 0.001). Additionally, at 5 months following the infection, the hospitalized patients with COVID-19 and prediabetes had a higher incidence of persistent diabetes mellitus compared to those without COVID-19. However, in non-hospitalized patients, those with and without COVID-19 and a history of prediabetes had similar rates of persistent diabetes mellitus [105]. Moreover, in a large national cohort of veterans analyzed in the United States, SARS-CoV-2 infection in males was found to be associated with a higher risk of developing incident diabetes compared to those who tested negative for the virus. In hospitalized participants, SARS-CoV-2 infection was associated with a higher risk of developing diabetes in males at both 10 days and at the end of the follow-up period (OR 1.40 (1.24–1.58) and 1.23 (1.12–1.36)). Conversely, among hospitalized females, there was no significant association between COVID-19 infection and the risk of developing diabetes [106]. Another comprehensive review revealed that the incidence of new-onset T1DM following SARS-CoV-2 infection ranged from 1.42 to 3.74, while the incidence of T2DM ranged from 1.30 to 2.71. To complete the epidemiological picture, they identified several risk factors, such as disease severity, age, ethnicity, mechanical ventilation and smoking habits, which were significantly associated with the development of DM following SARS-CoV-2 infection [107]. Indeed, the literature needs further investigation into the potential diabetogenic effect of COVID-19 and the precautions that may be taken into consideration. A summary of the papers mentioned above is available in Table 1.

Clearly, more research is needed to better establish the connection between the restrictions due to COVID-19 and the worsening status of type 2 diabetes cases. New-onset diabetes can present under the portrait of many diseases, and it can easily overlap with a multitude of other symptoms. Therefore, pediatricians must pay great attention to clinical findings and consider diabetes mellitus as a main diagnosis when having a new presentation with various symptoms. Recently, diabetes mellitus has also occurred in the setting of multisystem inflammatory syndrome in children and, therefore, glycemic monitoring should be considered in MIS-C management. Moreover, it is important that parents help their children to maintain healthy routines during the pandemic, such as wake-up routines, lunch schedules, exercises, online social time with friends as well as family time and reading before bed to limit the impact of COVID-19. According to literature data, diabetes carries a significant risk of morbidity and mortality (2.3% overall compared with 7.3% in patients with diabetes) [110] in patients with COVID-19, and it is of the utmost importance that children with diabetes pursue their vigilance, their hygiene routines and social distancing measures. Based on all the above and knowing that T2DM is linked to various comorbidities and a weakened immune system, which puts affected patients at a higher risk of COVID-19-related complications and mortality [111], it appears justifiable to suggest that obese patients, particularly those with a higher BMI, should be advised to receive COVID-19 vaccination [112]. Moreover, rigorously screening patients’ mental health status and necessities by health-care providers must not be neglected as the psychosocial distress can have an undesirable impact on diabetes management [113]. 

## 4. Conclusions

In general, it is evident that the impact of COVID-19 infection on children and young adults with DM is bidirectional. COVID-19 infection in children can have implications for those with underlying diabetes mellitus, encompassing both type 1 and type 2 diabetes. While the prevalence of severe COVID-19 is generally lower in children compared to adults, children with diabetes mellitus may be at an increased risk of experiencing severe illness and complications when infected with COVID-19. Also, SARS-CoV-2 infection is responsible for inducing newly diagnosed cases of diabetes, with an incidence of both T1DM and T2DM that has seemed to be increasing since the beginning of the pandemic. 

The relationship between COVID-19 infection and pediatric diabetes mellitus involves a complex interplay of immune dysregulation, inflammatory response, glycemic control disruption and potential pancreatic involvement, but further research is needed to elucidate the specific mechanism underlying this relationship and to inform optimal management strategies for this vulnerable population. Close collaboration between pediatric endocrinologists and infectious disease specialists is vital to ensure integrated care, proper infection control measures and timely interventions. 

## Figures and Tables

**Table 1:** Summary of the key papers regarding pediatric T1DM and T2DM and their main clinical findings.

Author	Year	Regions	Age (Years)	Study Period	Findings
Rabbone et al. [46]	2020	Italy	NA	2019–2020	Decrease in diabetes presentation and in severity of DKA
Dayal et al. [47]	2020	India	Pediatric population	April 2019–March 2020	Major reduction in hospitalization of children with onset of T1DM in April 2020
Brothers et al. [42]	2021	NA	12	NA	Sepsis due to Candida glabrata in a teenager with COVID-19 and T1DM
Williams et al. [48]	2021	United Kingdom	Pediatric population	1 January–31 July 2020	No evidence of diagnostic delay or increased illness severity for childhood cancer or T1DM
Ng et al. [108]	2020	United Kingdom	Pediatric population	1 March 2020–30 June 2020	An increased percentage of DKA was observed during the pandemic
Mianowska et al. [96]	2021	Poland	T1DM aged 8–18 years and their parents	November 2019–February 2020	COVID-19 lockdown did not seem to aggravate diabetes manifestations
Rahmati et al. [101]	2022	NA	T1DM pediatric patients	Up to March 2022	Notable increase in the worldwide incidence of pediatric new-onset T1DM, DKA, and severe DKA during the initial year of the pandemic
Lee et al. [81]	2022	South Korea	T1DM or T2DM patients <18 years	2018–2020	During the pandemic, the proportion of DKA cases increased compared to the pre-pandemic period
Wu et al. [109]	2021	China	T1DM pediatric patients	1 November 2019–31 July 2020	Glycemic control did not decrease in T1DM pediatric patients during the COVID-19 pandemic
Sadisharan Pillai et al. [77]	2023	United States	New-onset T2DM pediatric patients	1 January 2017–31 December 2021	The yearly occurrence of T2DM demonstrated an almost threefold rise during the pandemic compared to the preceding period

## Data Availability

No new data was generated.
